# Validation of a methylation-based signature for subventricular zone involvement in glioblastoma

**DOI:** 10.1007/s11060-024-04570-0

**Published:** 2024-02-20

**Authors:** Felix Ehret, Oliver Zühlke, Leonille Schweizer, Johannes Kahn, Christoph Csapo-Schmidt, Siyer Roohani, Daniel Zips, David Capper, Sebastian Adeberg, Amir Abdollahi, Maximilian Knoll, David Kaul

**Affiliations:** 1 Department of Radiation Oncology, Charité – Universitätsmedizin Berlin, Corporate Member of Freie Universität Berlin and Humboldt-Universität Zu Berlin, Berlin, Germany; 2 Charité – Universitätsmedizin Berlin, Berlin, Germany; German Cancer Consortium (DKTK), partner site Berlin, and German Cancer Research Center (DKFZ), Heidelberg, Germany; 3 Institute of Neurology (Edinger Institute), University Hospital Frankfurt, Goethe University, Frankfurt am Main, Germany; 4 Department of Radiology, Health and Medical University, Potsdam, Germany; 5 Department of Neuroradiology, Charité – Universitätsmedizin Berlin, Corporate Member of Freie Universität Berlin and Humboldt-Universität Zu Berlin, Berlin, Germany; 6 Berlin Institute of Health at Charité – Universitätsmedizin Berlin, BIH Biomedical Innovation Academy, BIH Charité Junior Clinician Scientist Program, Berlin, Germany; 7 Department of Neuropathology, Charité – Universitätsmedizin Berlin, Corporate Member of Freie Universität Berlin and Humboldt-Universität Zu Berlin, Berlin, Germany; 8 Department of Radiation Oncology, University Hospital Marburg/Gießen, Marburg, Germany; 9 Department of Radiation Oncology, Heidelberg University Hospital, Heidelberg, Germany

**Keywords:** Glioblastoma, IDH, Wild type, Subventricular zone, Methylation, Overall survival

## Abstract

**Purpose:**

Glioblastomas (GBM) with subventricular zone (SVZ) contact have previously been associated with a specific epigenetic fingerprint. We aim to validate a reported bulk methylation signature to determine SVZ contact.

**Methods:**

Methylation array analysis was performed on *IDHwt* GBM patients treated at our institution. The v11b4 classifier was used to ensure the inclusion of only receptor tyrosine kinase (RTK) I, II, and mesenchymal (MES) subtypes. Methylation-based assignment (SVZM ±) was performed using hierarchical cluster analysis. Magnetic resonance imaging (MRI) (T1ce) was independently reviewed for SVZ contact by three experienced readers.

**Results:**

Sixty-five of 70 samples were classified as RTK I, II, and MES. Full T1ce MRI-based rater consensus was observed in 54 cases, which were retained for further analysis. Epigenetic SVZM classification and SVZ were strongly associated (OR: 15.0, *p* = 0.003). Thirteen of fourteen differential CpGs were located in the previously described differentially methylated LRBA/MAB21L2 locus. SVZ + tumors were linked to shorter OS (hazard ratio (HR): 3.80, *p* = 0.02) than SVZM + at earlier time points (time-dependency of SVZM, *p* < 0.05). Considering the SVZ consensus as the ground truth, SVZM classification yields a sensitivity of 96.6%, specificity of 36.0%, positive predictive value (PPV) of 63.6%, and negative predictive value (NPV) of 90.0%.

**Conclusion:**

Herein, we validated the specific epigenetic signature in GBM in the vicinity of the SVZ and highlighted the importance of methylation of a part of the LRBA/MAB21L2 gene locus. Whether SVZM can replace MRI-based SVZ assignment as a prognostic and diagnostic tool will require prospective studies of large, homogeneous cohorts.

**Supplementary Information:**

The online version contains supplementary material available at 10.1007/s11060-024-04570-0.

## Introduction

Glioblastoma (GBM) is the most common malignant primary brain tumor in adults [1]. Despite trimodal treatment consisting of surgery, radiotherapy, and chemotherapy, the prognosis remains poor [2]. GBM may originate from self-renewing tumorigenic cancer stem cells (CSC) that rely on signals from their cellular milieu to maintain stem cell properties [3]. The subventricular zone (SVZ) is the most abundant location for neuronal stem cells (NSC) lining the lateral ventricles. It is actively involved in neurogenesis and may contribute to tumorigenesis in GBM. Tumors in this location may maintain a distinct immunosuppressed microenvironment [4–7]. A clonal relationship exists between driver mutations in SVZ tissue and GBM tissue, providing possible evidence that SVZ NSCs could be the cell of origin [5, 6, 8]. DNA methylation analysis has improved the molecular characterization of central nervous system tumors and led to the discovery of further tumor entities [9, 10]. GBM with SVZ contact might show a distinct molecular signature as they are closely related to a stem cell-rich zone that can also harbor GBM stem cells [3, 6, 11, 12]. Consequently, DNA methylation analysis and characterization could be important in understanding the role of the SVZ in GBM formation, self-renewal, and therapy resistance. A specific methylation signature recently reported distinguishes between GBM with and without SVZ involvement in isocitrate dehydrogenase (*IDH*) wild type (*IDH*wt) GBM, an approach that has the potential to improve patient risk stratification and to individualize future patient care [12, 13]. This study aimed to validate the accuracy and usefulness of a DNA methylation-based SVZ classifier (SVZM).

## Methods

Methylation array analysis of *IDH*wt GBM patients treated at our institution was performed with Illumina Infinium Methylation 850 k profiling arrays (Illumina Inc., San Diego, CA, USA). Further classification of methylation profiles was done using the v11b4 classifier from the German Cancer Research Center (DKFZ, Heidelberg, Germany). Only receptor tyrosine kinase (RTK) I, II, and mesenchymal (MES) GBM subtypes were included. Treatment, molecular, and histopathological data were obtained from the patients’ medical records and institutional databases. Preoperative magnetic resonance imaging (MRI) was independently reviewed by three experienced readers, two neuroradiologists, and one radiation oncologist (JK, CCS, FE), who assessed GBM SVZ contact on T1-weighted contrast-enhanced MRI (T1ce) scored as a binary variable (SVZ contact [SVZ +] vs. no contact [SVZ-]). Readers were mutually blinded to each other's assessments. Tumor contact with the SVZ was defined as T1ce tumor lesions being present within a 5 mm area adjacent to the lateral ventricles. Patients were labeled as SVZ + or SVZ- when all three readers unanimously agreed on the SVZ contact assessment, and only these samples were retained for subsequent analysis. Contrast-enhancing tumor lesion volume on T1ce and the shortest distance from the outline of the contrast-enhancing GBM to the SVZ were assessed in the EclipseTM treatment planning system (Varian Medical Systems Inc., Palo Alto, CA, USA). A consensual SVZ contact assessment based on MRI was used to validate the SVZM signature [12]. Methylation data was analyzed using the minfi package in R [14]. Idat files were normalized using minfis’ preprocessFunnorm() function, subsequent analyses were performed with M-values (log2 ratio of the methylated versus unmethylated probe intensities). As previously described, the 15 CpG SVZM signature was used to assign samples using hierarchical cluster analysis (Euclidean distance, average linkage) by identifying two main clusters [12]. Manual comparison of overall cluster methylation patterns and of mean methylation of the 15 CpG signature was used to define clusters as SVZM + and SVZM- (Suppl. Fig. 1). Differential methylation between SVZM ± samples was tested using the *tTest* function from the dataAnalysisMisc package. P-values were adjusted for multiplicity using the Bonferroni method [15]. We used a significance level α of 0.05. We compared the molecular SVZM signature to our consensual T1ce SVZ assessment using Fisher’s exact test, and calculated sensitivity, specificity, positive predictive value (PPV), and negative predictive values (NPV).

Overall survival (OS) was calculated from the date of surgery to the date of death by any cause. Progression-free survival (PFS) was calculated from the date of surgery to tumor progression determined by MRI or death by any reason. Censoring for OS and PFS occurred without prespecified events at the last available follow-up. Univariable analyses were performed with Cox-PH models (*survival* package) [16]. The proportionality assumption was evaluated visually for identity (time), log(time), and log(time + offset) (offset: = 10) transformations (Suppl. Fig. 2), and tested using the *cox.zph* function. For time-dependent coefficients, we used the *time-transform* (*tt*) functionality in Cox models. This study was approved by the local institutional review board (EA1/061/22).

## Results

### Patient characteristics

**Fig. 1:**
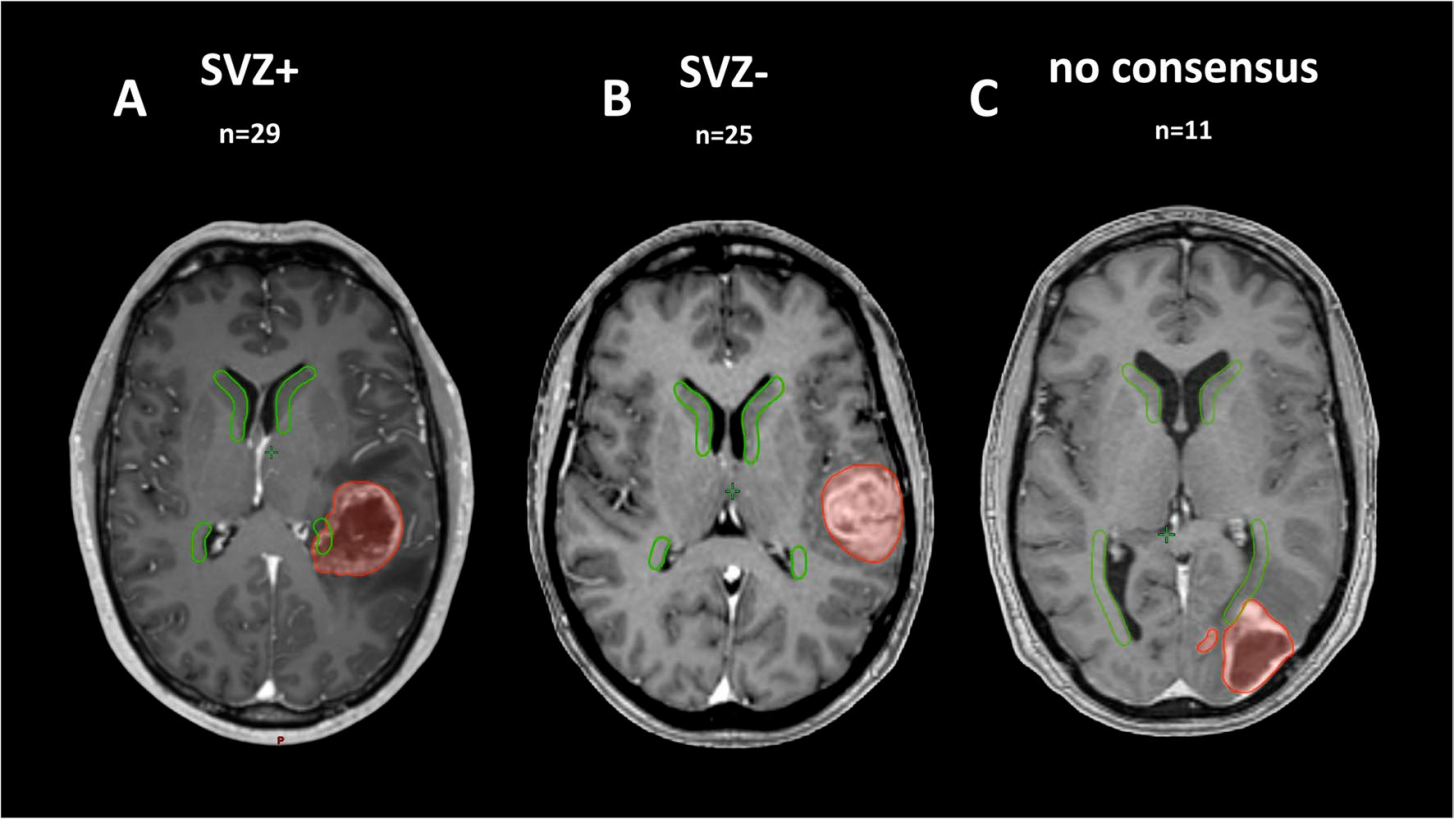
Representative axial T1ce MRI for SVZ contact assessment and three examples. **A** SVZ + , **B** SVZ-, and (**C**) missing consensus amongst readers. SVZ = green, GBM = red

We identified and analyzed 70 *IDH*wt GBM patients that underwent treatment between 2006 and 2021. Sixty-five of the samples were classified as MES (*n* = 23)/RTK I (*n* = 16)/RTK II (*n* = 26) GBM using the brain tumor classifier v11b4, and only these were retained for subsequent analysis [10]. In 54 of 65 cases (83.1%), all three independent viewers unanimously agreed on either contact or no contact with the SVZ (Fig. 1A-B). Eleven cases (16.9%) without agreement (Fig. 1C) were excluded from the analysis. Baseline characteristics for the 54 included patients are shown in Table 1.

**Table 1:** Patient, tumor, and treatment characteristics of cases with consensus assessment of SVZ contact

*IDHwt* GBM	Total cohort		SVZ +		SVZ-		SVZM +		SVZM-	
	*n* = 54	%	*n* = 29	%	*n* = 25	%	*n* = 44	%	*n* = 10	%
Sex										
Male	31	57.4%	20	69.0%	11	44.0%	26	59.1%	5	50.0%
Female	23	42.6%	9	31.0%	14	56.0%	18	40.9%	5	50.0%
Age in years										
Mean	57		58		56		58		53	
Min	35		35		36		35		39	
Max	81		81		75		81		74	
Surgery										
Total resection	32	59.3%	14	48.3%	18	72.0%	25	56.8%	7	70.0%
Subtotal resection	6	11.1%	3	10.3%	3	12.0%	4	9.1%	2	20.0%
Biopsy	16	29.6%	12	41.4%	4	16.0%	15	34.1%	1	10.0%
RCT										
Yes	39	72.2%	21	72.4%	18	72.0%	30	68.2%	9	90.0%
No	4	7.4%	3	10.3%	1	4.0%	3	6.8%	1	10.0%
N/A	11	20.4%	5	17.2%	6	24.0%	11	25.0%	0	0.0%
Adjuvant TMZ										
Yes	41	75.9%	22	75.9%	19	76.0%	35	79.5%	6	60.0%
No	4	7.4%	3	10.3%	1	4.0%	3	6.8%	1	10.0%
N/A	9	16.7%	4	13.8%	5	20.0%	6	13.6%	3	30.0%
Contrast enhancing										
Tumor volume (T1ce MRI) in cm^3^										
Median	22.9		51.8		5.5		34.0		16.4	
Min	0.1		1.7		0.1		0.1		0.1	
Max	114.6		114.6		55.1		14.6		63.9	
Multifocal disease										
Yes	14	25.9%	10	34.5%	4	16.0%	11	25.0%	3	30.0%
No	40	74.1%	19	65.5%	21	84.0%	33	75.0%	7	70.0%
Methylation classifier v11b4										
GBM MES	20	37.0%	11	37.9%	9	36.0%	18	40.9%	2	20.0%
GBM RTK 1	13	24.1%	11	37.9%	2	8.0%	11	25.0%	2	20.0%
GBM RTK2	21	38.9%	7	24.1%	14	56.0%	15	34.1%	6	60.0%
MGMT promotor methylation										
Yes	22	40.7%	9	31.0%	13	52.0%	17	38.6%	5	50.0%
No	31	57.4%	20	69.0%	11	44.0%	27	61.4%	4	40.0%
N/A	1	1.9%	0	0.0%	1	4.0%	0	0.0%	1	10.0%
KPS										
≥ 80	41	75.9%	21	72.4%	20	80.0%	32	72.7%	9	90.0%
< 80	10	18.5%	7	24.1%	3	12.0%	9	20.5%	1	10.0%
N/A	3	5.6%	1	3.4%	2	8.0%	3	6.8%	0	0.0%

*IDHwt GBM* Isocitrate dehydrogenase wildtype glioblastoma; *SVZ* Subventricular zone; *SVZ* + subventricular zone contact judged by the raters; *SVZM* + Methylation-predicted subventricular zone contact; *n* Sample size; *RCT* Radiochemotherapy; *TMZ* Temozolomide; *N/A* Not available; *T1ce MRI* T1-weighted contrast-enhanced magnetic resonance imaging; *cm*^*3*^ Cubic centimeter; *GBM MES* Mesenchymal glioblastoma; *GBM RTK 1* Glioblastoma receptor tyrosine kinase 1; *GBM RTK2* Glioblastoma receptor tyrosine kinase 2; *MGMT* O^6^-methylguanine-DNA methyltransferase; *KPS* Karnofsky performance status

Thirty-two patients had gross total resections (59.3%), six subtotal resections (11.1%), and 16 a biopsy (29.6%). Thirty-nine patients (72.2%) underwent radiochemotherapy, and 41 patients (75.9%) received chemotherapy with temozolomide (TMZ). Median contrast-enhancing tumor volume was 22.9 cm^*3*^ (range: 0.1 cm^*3*^ to 114.6 cm^*3*^). GBM were mainly located in the frontal (17 patients, 31.5%), followed by temporal (twelve patients, 22.2%), and parietal lobes (five patients, 9.3%). Fourteen patients (25.9%) had multifocal disease at diagnosis. Twenty-two (40.7%) tumors showed O^6^-methylguanine-DNA-methyltransferase (*MGMT*) promoter methylation.

### Image and methylation-based classification

**Fig. 2:**
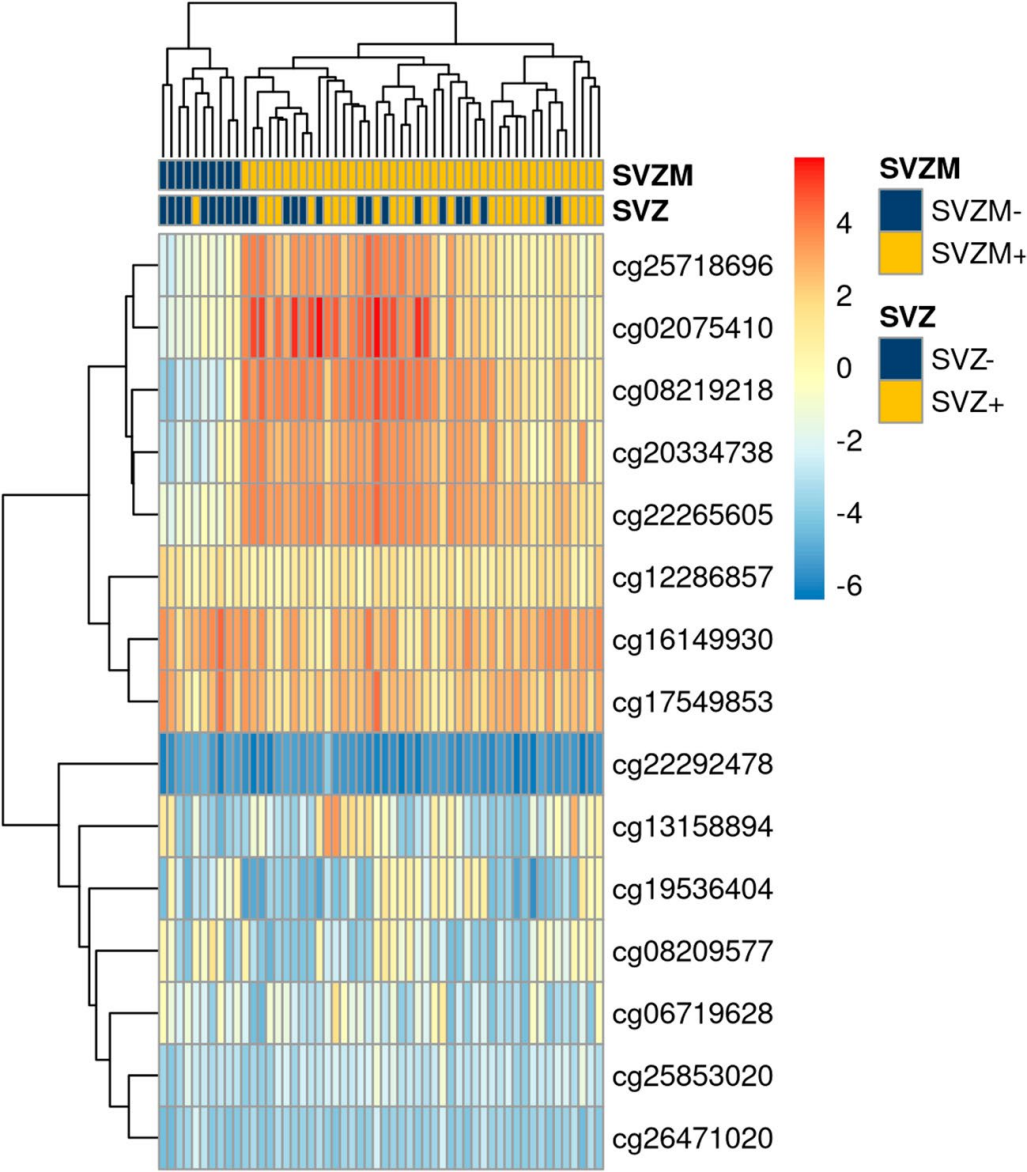
Clustering of the 15 CpG signature SVZM versus T1ce MRI assessment. M-values visualized as blue/hypomethylated (-6) to red/hypermethylated (+ 6). The columns represent one patient assigned to the SVZM + or SVZM- based on hierarchical clustering. Each row depicts a CpG

Twenty-nine cases (53.7%) were classified as SVZ + , and 25 (46.3%) cases as SVZ-. Patients with SVZ + assigned tumors showed larger contrast-enhancing tumor volume (median 51.8 vs. 5.5 cm^3^) and a higher fraction of Karnofsky performance status (KPS) < 80% (*n* = 7, 24.1% vs *n* = 3, 12.0%), MGMT promoter methylation was less common (*n* = 9, 31.0% vs. *n* = 13, 52.0%). Figure 2. shows the hierarchical clustering of M-values of the previously reported 15 CpG SVZM signature used for SVZM classification. The proportion of SVZ ± samples was balanced (*n* = 29, 53.7% vs *n* = 25, 46.2%). Considering SVZM, only 18.5% (*n* = 10) of samples were classified as SVZM-.

**Fig. 3:**
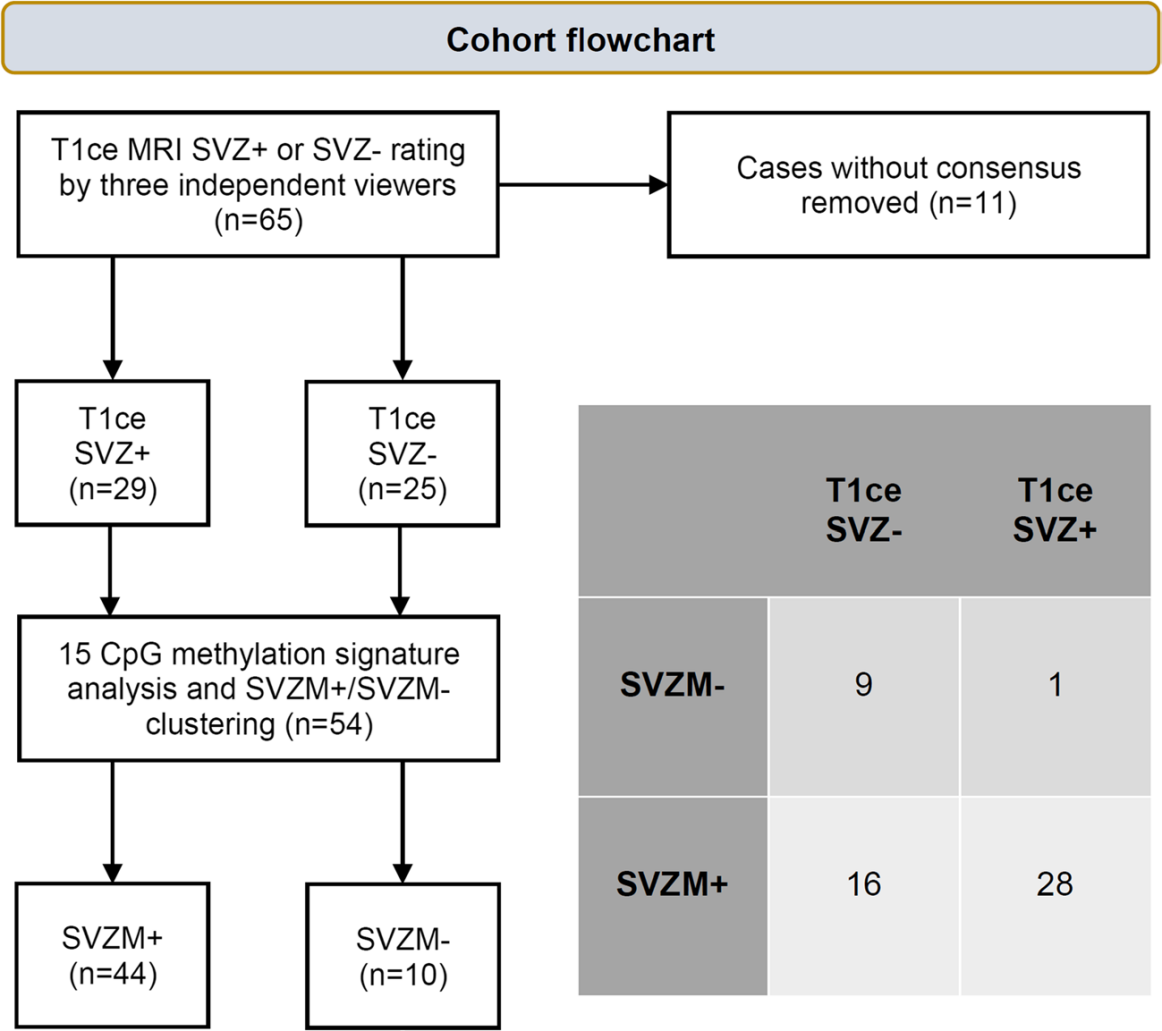
Identification of the patient cohort and SVZ/SVZM results. Fishers’ exact test indicated a strong association between MRI-based SVZ classification and SVZM with an odds ratio (OR) of 15.0 (95% CI: 1.8–711.5, *p* = 0.003)

Fisher’s exact test indicated a strong association between MRI-based SVZ classification and SVZM with an odds ratio (OR) of 15.0 (95% confidence interval (CI): 1.8–711.5, *p* = 0.003) (Fig. 3). This corresponds to 28 true positives (TP), 16 false positives (FP), one false negative (FN), and nine true negatives (TN) if consensus SVZ classification is considered the ground truth. This leads to a sensitivity of 96.6%, specificity of 36.0%, PPV of 63.6%, and NPV of 90.0% for SVZM.

We further assessed which CpGs are most strongly correlated with SVZ/SVZM assignment. We found no CpGs associated with SVZ for a conservative p-value adjustment but 14 CpGs associated with SVZM. Thirteen out of these 14 CpGs were located on the LRBA/MAB21L2 locus, including all 4 LRBA/MAB21L2 CpGs from the 15 CpG signature (Suppl. Fig. 3). This underlines the role of the LRBA/MAB21L2 locus on chr 4 (pos 151,502,935–151,505,084; hg19) for the classification signature.

### Prognostic value of SVZ and SVZM

**Fig. 4:**
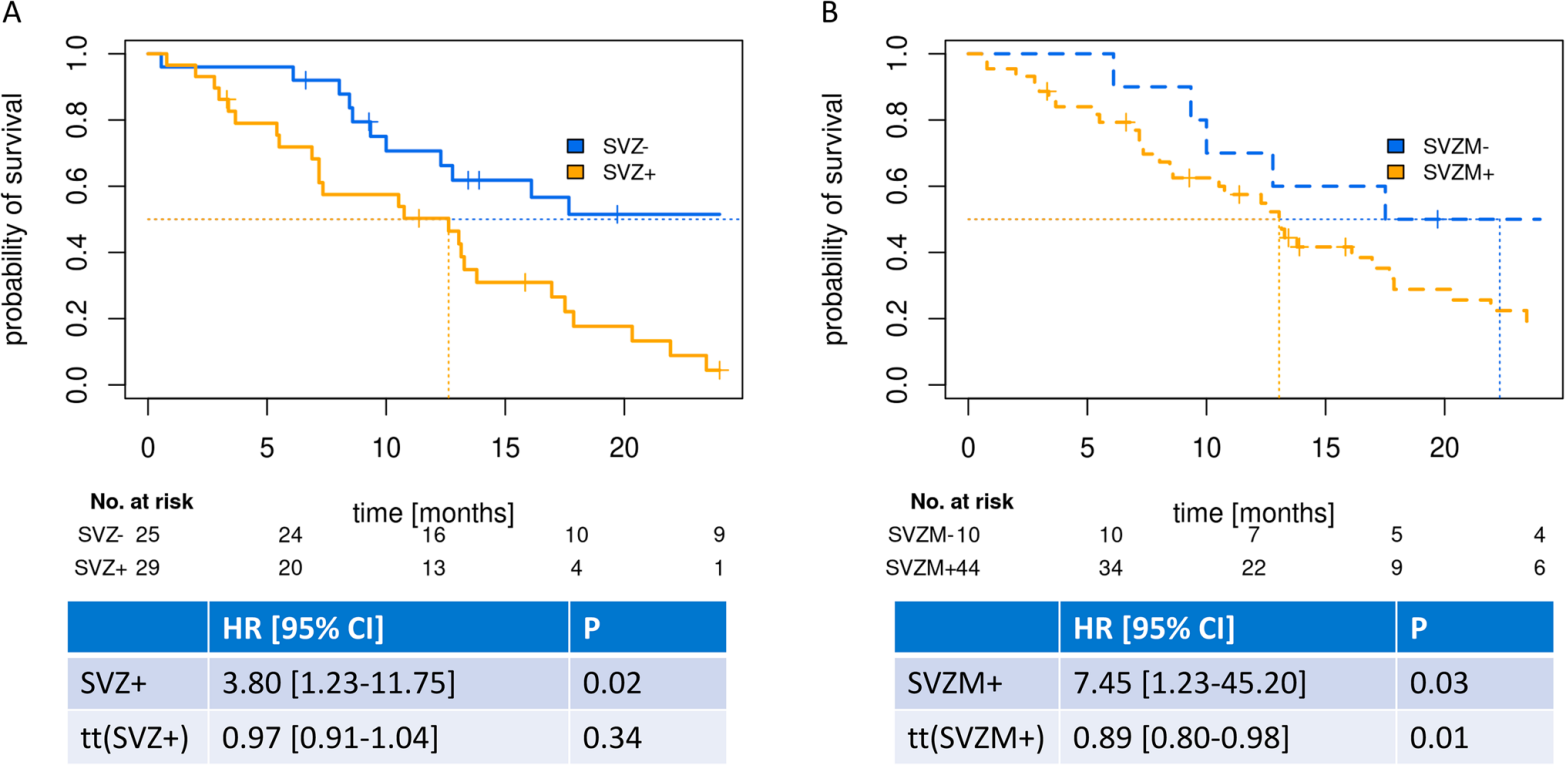
**a** Overall survival stratified by SVZ contact, **b** Overall survival stratified by the methylation-based signature

We evaluated the prognostic value of SVZ and SVZM for OS in univariable analyses (Fig. 4). We observed non-proportionality of hazards for SVZM but not for SVZ (Suppl. Fig. 2), indicating a time-dependency of hazards. Patients with SVZ + consensus-classified tumors had shorter survival times (hazard ratio (HR) 3.80 [1.23–11.75], *p* = 0.02) than patients with SVZM + classified tumors (SVZM + : HR 7.45 [1.23–45.20], *p* = 0.03), especially at earlier time points (tt(SVZM +): 0.89 [0.80–0.98], *p* = 0.01). Median OS was comparable for SVZ + (12.6 months, 95% CI [7.18–17.5]) and SVZM + (13.0 months, 95% CI [8.59–17.9]). At two years, no patients from the SVZ + group (0%, 95%CI [0–11.7]) and two patients (4.5%, 95% CI [0.13–15.14]) from the SVZM + group were still alive (trend towards a better separation of SVZ +). Next, we performed a univariable analysis of PFS (Suppl. Fig. 4). For SVZ, no prognostic separation could be observed, for SVZM- patients, a tendency towards longer PFS was observed (*p* = 0.09).

## Discussion

Herein, we report the validation results of a previously established methylation-based signature for SVZ contact of GBM [12]. To our knowledge, this is the first attempt to validate the reported SVZ methylation signature. The first step to verify the molecular classifier was the imaging assessment of SVZ contact. We chose a multi-reader, interdisciplinary approach to reduce misclassification biases. However, eleven cases (16.9%) had no agreement on SVZ contact. This is partly due to the definition of the SVZ, which, while formally standardized, still holds the potential for differing interpretation. Precise measurement is crucial as only a few millimeters differences can determine SVZ contact. Adeberg et al. also experienced a variability of 38% in the validation process of their findings [12]. However, only one rater assessed the imaging at three different time points, indicating an intraobserver variability in contrast to our interobserver variability. Such differences in MRI-based GBM SVZ contact assessment have been previously described, but the clinical implications are still ambiguous and need further clarification [13, 17].

In the neuroradiological assessment of high-grade gliomas usually two- or even three neuroradiologists assess the available imaging and find a consensus on tumor location and its spatial extension. This helps to improve the accuracy, consistency, and reliability of radiological assessment of gliomas. In addition, advanced imaging techniques such as diffusion tensor imaging, perfusion-weighted imaging, magnetic resonance spectroscopy, and positron emission tomography-computed tomography are becoming more regularly used. They can provide additional information about the spatial configuration of tumors [18]. Nevertheless, more precise and reliable methods are necessary to determine SVZ contact, given its potential role for risk stratification and future individualization of patient care [13, 19].

Next, we validated a previously described methylation-based signature to determine the tumors’ SVZ involvement and its role for OS [12]. The association between the MRI T1ce imaging rater assessment and SVZM was solid, with the signature showing a considerable performance concerning sensitivity and NPV. Moreover, the OS was reduced for T1-ce SVZ + and SVZM + compared to T1ce SVZ- and SVZM-. For methylation-based classification, we observed a prognostic separation, especially within the first year following diagnosis. Median OS was comparable between SVZ + (12.6 months) and SVZM + (13.0 months), hinting towards comparable prognostic separation. Consensus SVZ classification was time-independent – in contrast to SVZM – and thus conferred a more robust readout for this cohort. One can speculate that the close connection to the SVZ is indeed what enables these GBM to present such aggressive phenotypes. Besides, the SVZM signature yielded a high sensitivity and NPV, underlining the potential to utilize it as a screening tool for SVZ contact and to confirm the absence of it in case of corresponding imaging findings.

In our cohort, SVZM classified 81% of tumors as SVZ-associated, whereas in the original study, SVZM positive and negative GBM were equally distributed [12]. In most published studies that concentrate on MRI-based SVZ definition, the proportions of participants belonging to the SVZ-positive and SVZ-negative groups were relatively comparable, ranging from 54 to 70% [20, 21]. Barami et al. mentioned that 93 out of 100 of low- to high-grade glioma patients (53 GBM) had shown contact with the lateral ventricular wall based on MRI [22]. It is worth noting that in these studies, the criterion for defining SVZ contact was based on lateral contact with the lateral ventricles rather than contact with a 5 mm margin adjacent to the lateral ventricles. However, our findings suggest that a substantial proportion of GBM might be associated with the SVZ, with the limitation of potential sampling biases.

Adeberg et al. described an “*epigenetic and transcriptional silencing of MAB21L2/LRBA in SVZM* + *tumors* “, possibly resulting in transforming growth factor β activity and a dysregulated immune response. We were able to confirm the LRBA/BA21L2 locus as an important component of the SVZM signature. Adeberg et al. also noted a hypomethylation resulting in increased gene expression for 90% of the CpG that defines SVZM + [12]. It must be noted that some authors have reported that SVZ contact is not associated with any molecular signature, including methylation, while other authors have suggested specific changes in gene expression levels of GBMs with SVZ contact [23, 24]. Furthermore, a relationship may exist between driver mutations in both the healthy SVZ tissue and tumor tissue, suggesting that SVZ NSCs might be the source of GBM [6, 8]. Another area of interest is the role of cerebrospinal fluid (CSF) in GBM formation. As the SVZ is located close to the cerebrospinal fluid compartment, tumors contacting the SVZ could experience changes in their microenvironment and hence develop an altered methylation status. CSF has been shown to promote tumorigenic capacities in vitro and tumor growth in vivo and to cause transcriptomic changes in human GBM, leading to higher malignancy [25]. The altered DNA methylation signature in SVZ + tumors could possibly be connected to CSF exposure.

In our patient cohort, we noted that SVZ involvement was accompanied by a trend of larger tumor volume, worse KPS, and a higher incidence of multifocal tumor spread, which may all have negative implications for the observed OS (Suppl. Fig. 5). A plausible explanation for this observation is that larger tumors have a higher likelihood of contacting a specific area, in our case, the SVZ. Furthermore, the feasibility of surgical removal is hampered in large tumors where critical structures are more likely to be involved, which in turn results in worse performance status. Finally, infiltration of the ventricles is another factor that may occur in cases with SVZ contact, potentially exerting a negative influence on survival.

In our cohort, tumors with MRI-based SVZ involvement had a higher likelihood of presenting as multifocal at the date of diagnosis. This observation was also made by Lim et al., where 40% of SVZ-associated GBM presented as multifocal compared to 14% in the SVZ- group. Ahmadipour et al. showed similar results in their cohort in which 49% of SVZ + GBM were multifocal compared to 18% in the SVZ- group [26, 27]. Various studies have assessed the role of SVZ contact as a prognostic factor, with many of them confirming its negative association with survival [13, 28, 29].

DNA methylation analysis and the use of large-scale methylation arrays have enabled the classification of CNS tumors into different subtypes [10]. There is an ongoing search for genetic and epigenetic signatures that can help us to further understand the complex mechanisms involved in GBM formation. The described DNA methylome-based classifier of SVZ contact may be a valuable asset to improve diagnostic accuracy and patient stratification. This validation analysis provides further evidence on the potential utility of methylation signatures to improve patient and tumor stratification. Nevertheless, further evaluation with a higher number of patients who clearly show no signs of radiological SVZ involvement is needed to further validate its prognostic role.

This work has some limitations that should be considered when interpreting the results. The low number of cases that show no signs of SVZ involvement limits the generalizability of our findings. Multivariable analysis was limited due to the small number of patients and events. A much larger and more homogeneous patient population with balanced baseline characteristics and prospective study design is warranted to ultimately determine the validity of the DNA methylation-assisted classification of SVZ involvement in GBM. Finally, future research should also investigate ways to improve and tweak the SVZ signature given the potential for further refinement.

## Conclusion

The reported and tested DNA methylation-based SVZ signature could become a valuable tool in advancing the molecularly and spatial characterization of GBM and may reduce inter- and intraobserver variability in the assessment of SVZ involvement. Its value as a prognostic marker warrants further studies with larger and more homogenous patient cohorts.

### Supplementary Information

Below is the link to the electronic supplementary material.

**Suppl. Figure 1.:** Mean methylation of 15CpG used for SVZM ± cluster assignment. p-value: linear model analysis. (JPG 146 kb)

**Suppl. Figure 2.:**  Evaluation of time dependency of covariates SVZM (upper row) and SVZ (bottom row) for the following transformations: identity(time), log(time), log(time + offset), offset: = 10. Plots: estimate of the time-dependent coefficient (cox.zph); scaled Schoenfeld residuals. For proportional hazards, fits would correspond to a horizontal line. p-values calculated with cox.zph. (JPG 832 kb)           

**Suppl. Figure 3.:**  Left: all CpGs annotated with LRBA and/or MAB21L2. Right: Differential CpGs between SVZM ± , Bonferroni adjusted p -value < 0.05, t-test. Samples ordered by mean methylation. Rows ordered by position on chromosome. (JPG 2.49 mb)

**Suppl. Figure 4.: a)** Progression-free survival stratified by SVZ contact **b)** Progression-free survival stratified by the methylation-based signature. (JPG 5.19 mb)

**Suppl. Figure 5.:** T1ce based tumor volumes depending on SVZ/SVZM assignment. Left: values ranked by volume. Right: distribution by SVZ and SVZM. In four cases volume was not measurable due to a software error. Linear model p-value (Wald type). Red: mean values, with bootstrapped confidence limits. (JPG 1.22 mb)

## Data Availability

The datasets generated and analyzed during the current study are not publicly available.
