# Supplementary figures and images for: Validation of a methylation-based signature for subventricular zone involvement in glioblastoma
Source: J Neurooncol. 2024 Feb 20;167(1):89–97. doi: 10.1007/s11060-024-04570-0 (PMC10978677; doi:10.1007/s11060-024-04570-0)

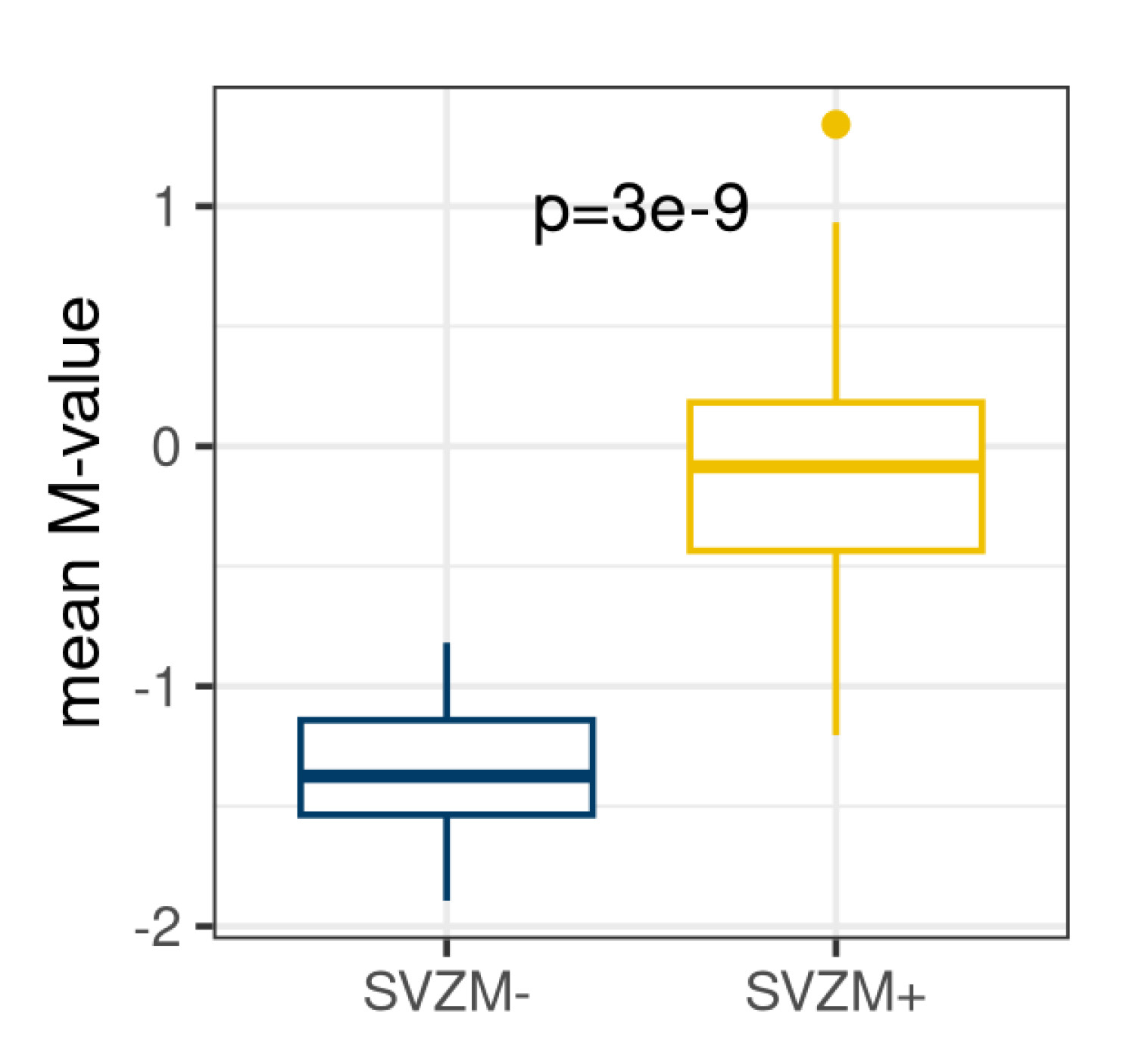

Supplement: Supplementary file 1 — Suppl. Figure 1.: Mean methylation of 15CpG used for SVZM ± cluster assignment. p-value: linear model analysis. (JPG 146 kb) [file 11060_2024_4570_MOESM1_ESM.jpg]

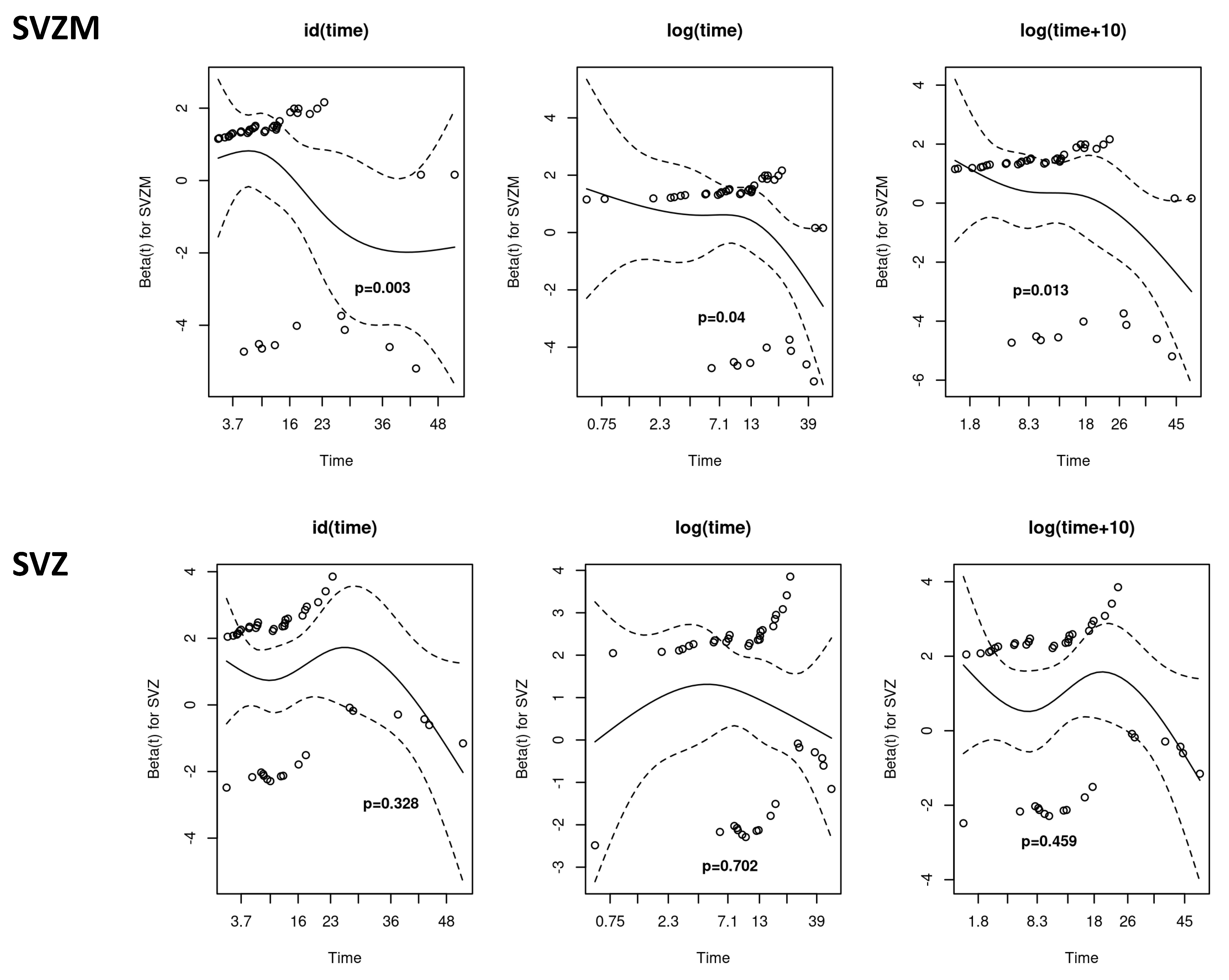

Supplement: Supplementary file 2 — Suppl. Figure 2.: Evaluation of time dependency of covariates SVZM (upper row) and SVZ (bottom row) for the following transformations: identity(time), log(time), log(time + offset), offset: = 10. Plots: estimate of the time-dependent coefficient (cox.zph); scaled Schoenfeld residuals. For proportional hazards, fits would correspond to a horizontal line. p-values calculated with cox.zph. (JPG 832 kb) [file 11060_2024_4570_MOESM2_ESM.jpg]

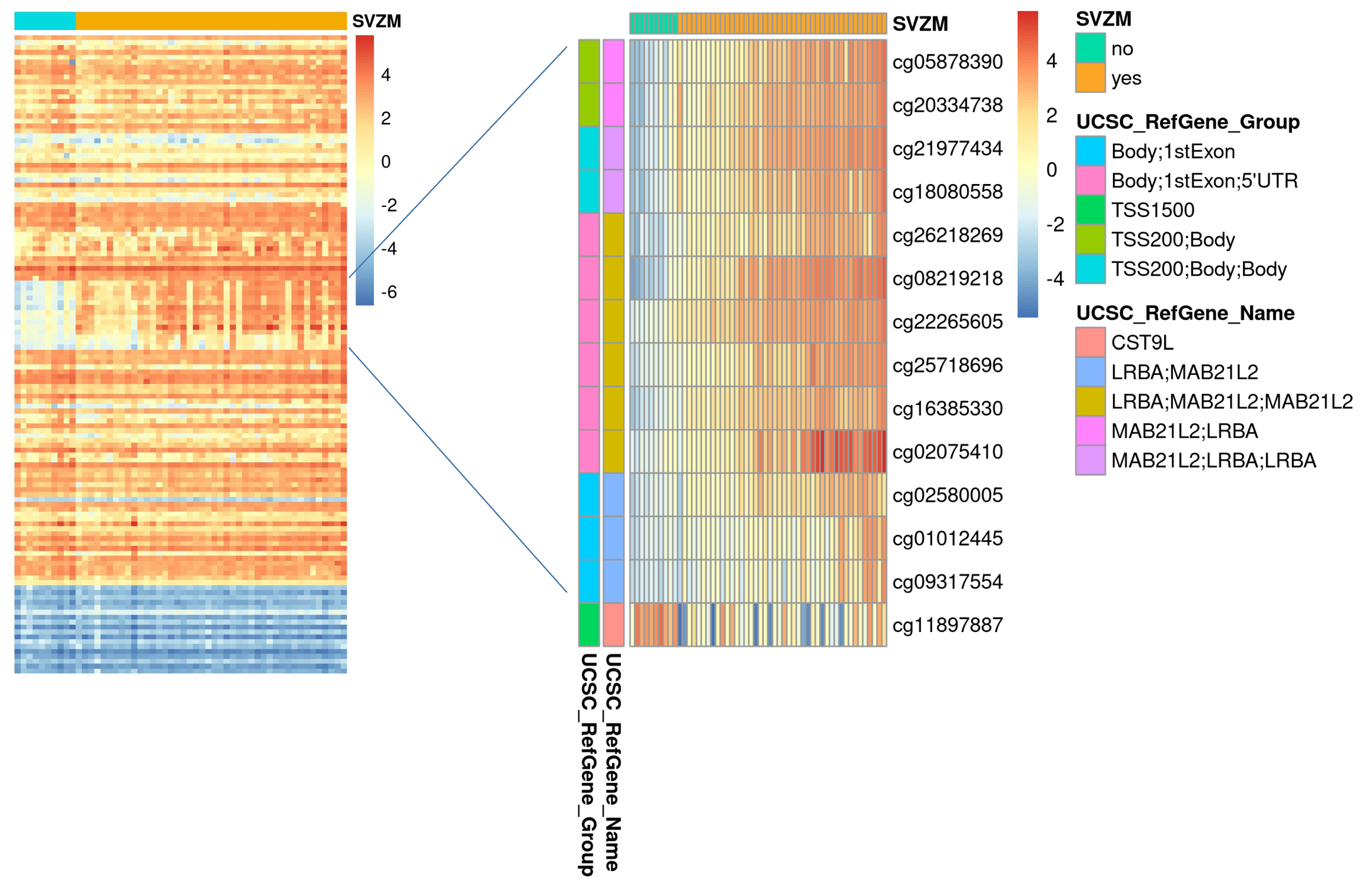

Supplement: Supplementary file 3 — Suppl. Figure 3.: Left: all CpGs annotated with LRBA and/or MAB21L2. Right: Differential CpGs between SVZM ± , Bonferroni adjusted p -value < 0.05, t-test. Samples ordered by mean methylation. Rows ordered by position on chromosome. (JPG 2.49 mb) [file 11060_2024_4570_MOESM3_ESM.jpg]

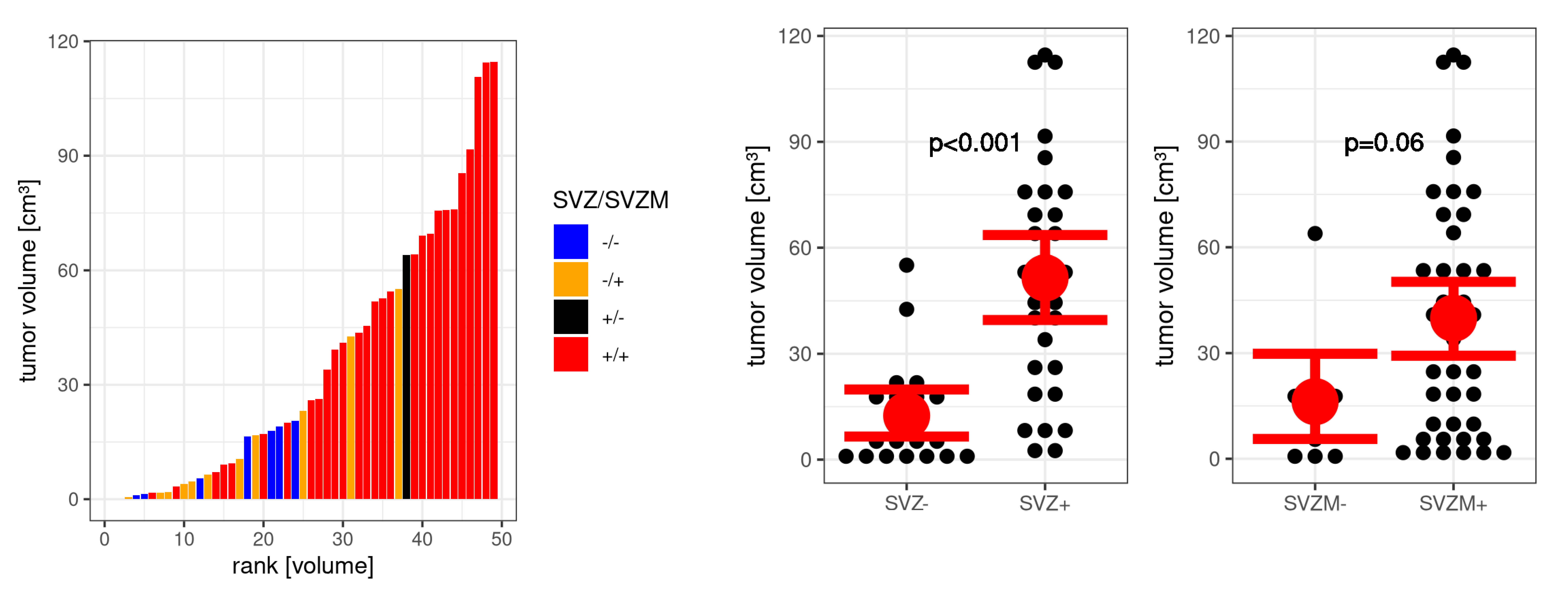

Supplement: Supplementary file 5 — Suppl. Figure 5.: T1ce based tumor volumes depending on SVZ/SVZM assignment. Left: values ranked by volume. Right: distribution by SVZ and SVZM. In four cases volume was not measurable due to a software error. Linear model p-value (Wald type). Red: mean values, with bootstrapped confidence limits. (JPG 1.22 mb) [file 11060_2024_4570_MOESM5_ESM.jpg]
